# Case–control study of leptospirosis in Aotearoa New Zealand reveals behavioural, occupational, and environmental risk factors

**DOI:** 10.1017/S0950268825100071

**Published:** 2025-06-02

**Authors:** Shahista Nisa, Enrico Ortolani, Emilie Vallée, Jonathan Marshall, Julie Collins-Emerson, Polly Yeung, Gerard Prinsen, Jackie Wright, Tanya Quin, Ahmed Fayaz, Stuart Littlejohn, Michael G. Baker, Jeroen Douwes, Jackie Benschop

**Affiliations:** 1 Molecular Epidemiology and Public Health Laboratory, School of Veterinary Science, Massey University, Palmerston North, New Zealand; 2 EpiCentre, School of Veterinary Science, Massey University, Palmerston North, New Zealand; 3 School of Mathematical and Computational Sciences, Massey University, Palmerston North, New Zealand; 4 School of Social Work, Massey University, Palmerston North, New Zealand; 5 School of People, Environment and Planning, Massey University, Palmerston North, New Zealand; 6 Institute of Environmental Science and Research, Health Security, Christchurch, New Zealand; 7 Rural Health Unit, University of Auckland, Auckland, New Zealand; 8 Department of Public Health, University of Otago, Wellington, New Zealand; 9 Centre for Public Health Research, Massey University, Wellington, New Zealand

**Keywords:** Leptospirosis, Zoonotic, Rodent, Livestock, Water

## Abstract

Leptospirosis in NZ has historically been associated with male workers in livestock industries; however, the disease epidemiology is changing. This study identified risk factors amid these shifts. Participants (95 cases:300 controls) were recruited nationwide between 22 July 2019 and 31 January 2022, and controls were frequency-matched by sex (90% male) and rurality (65% rural). Multivariable logistic regression models, adjusted for sex, rurality, age, and season—with one model additionally including occupational sector—identified risk factors including contact with dairy cattle (aOR 2.5; CI: 1.0–6.0), activities with beef cattle (aOR 3.0; 95% CI: 1.1–8.2), cleaning urine/faeces from yard surfaces (aOR 3.9; 95% CI: 1.5–10.3), uncovered cuts/scratches (aOR 4.6; 95% CI: 1.9–11.7), evidence of rodents (aOR 2.2; 95% CI: 1.0–5.0), and work water supply from multiple sources—especially creeks/streams (aOR 7.8; 95% CI: 1.5–45.1) or roof-collected rainwater (aOR 6.6; 95% CI: 1.4–33.7). When adjusted for occupational sector, risk factors remained significant except for contact with dairy cattle, and slaughter without gloves emerged as a risk (aOR 3.3; 95% CI: 0.9–12.9). This study highlights novel behavioural factors, such as uncovered cuts and inconsistent glove use, alongside environmental risks from rodents and natural water sources.

## Introduction

Leptospirosis is a neglected zoonotic disease that can cause a wide range of symptoms from mild flu-like illness to death [1]. Globally, approximately 1 million cases and 60 000 deaths occur annually [2], with a loss of 2.9 million disability-adjusted life years [3]. At-risk populations vary by location and include people who are in direct contact with infected animal urine or are indirectly exposed through contaminated environments, for example, contact with livestock (farmers and meat workers), contact with rodents (sewage workers, rice paddy workers, subsistence farmers, and urban slum dwellers), and contact with recreational and flood waters [4]. The highest burden of leptospirosis occurs in resource-poor tropical countries with incidence greater than 10/100 000 compared to high-income temperate countries where incidences range between 0.1 and 10/100 000 [5]. Aotearoa has one of the highest leptospirosis morbidities for a high-income temperate country [6], even though the estimates for incidence [7] and burden are under-ascertained [8] (human notification is compulsory under the New Zealand Health Act 1956 [9]).

Since the early 1970s, leptospirosis has been considered an occupational disease in Aotearoa when the incidence peaked at 30/100 000, with abattoir workers, dairy farmers, and pig farmers identified as high-risk occupations [10]. From the 1980s, cattle and pig vaccines were developed, and a combination of animal vaccination programmes [11, 12], guidelines on dairy farm operations [13], and personal protective equipment (PPE) among agricultural workers were implemented to prevent human infections. While incidence has decreased to 2.0/100 000 over time (1999–2017), these interventions have been only partially effective. Analysis of human notification data revealed infections from serovars not included in current animal vaccines [14, 15] and, the use of protective equipment did not necessarily prevent infection [16]. Furthermore, from 2017 to 2019, there was an 89% increase in cases compared with the previous 5 years (2012–2016), and there has been an increase in cases from occupations traditionally not considered high-risk [14, 17]. These emerging trends suggest knowledge gaps on current risk factors.

We hypothesize that the shifting disease patterns indicate a transition towards a more tropical, epidemic-style transmission of leptospirosis in Aotearoa, with rodent and environmental pathways becoming increasingly important. The overall aim of this study was to identify modifiable risk factors for human leptospirosis in Aotearoa to inform effective policies and practices to lower the incidence and the associated social and economic burden.

## Methods

### Study design

This was a nationwide case–control study frequency-matched by sex and rurality, with a 2:1 ratio of controls to cases, with the aim of recruiting 300 controls and 150 cases in Aotearoa. For common exposures (prevalence 30%–70%) [18], this would provide >80% power for odds ratios (ORs) as low as 1.8, while for less common exposures (15%) [19], this would provide 80% power to detect ORs as low as 2.1. The matching frequency was based on the distribution of sex and rurality of leptospirosis notifications in Aotearoa from 1 January 2014 to 31 December 2018, which included 90% males and 65% living in rural areas [20]. Rurality was determined by home address according to the most recent urban:rural classification method by Statistics New Zealand [21]. Participants were recruited between 22 July 2019 and 31 January 2022. Full details of the study design and methods, including case and control definitions, recruitment strategy, data collection, and questionnaire development, have been published in a protocol paper [22].

### Study participants

The initial case definition included individuals who met the New Zealand Ministry of Health definition for a confirmed or probable leptospirosis case [23]. The case definition was expanded twice during the study based on data from one of the two diagnostic laboratories that perform serological tests. These data revealed that many cases were under-ascertained between 2019 and 2020 as 72% (540/747) of patients suspected of leptospirosis did not return to provide the required second sample for leptospirosis serological testing and were therefore never diagnosed. Thus, the case definition was expanded to include patients who tested positive: (a) on any one sample, including an Immunoglobulin M screening test in diagnostic laboratories from 15 October 2020 or( b) by polymerase chain reaction in the research laboratory (Molecular Epidemiology and Public Health Laboratory) from 28 January 2021.

Controls consisted of participants from the 2016/2017 and 2017/2018 New Zealand Health Surveys (NZHS) [24] that had previously agreed to be approached for future surveys. Potential controls were excluded if they reported experiencing an influenza-like illness in the 4 weeks preceding the control survey to reduce the chances of enrolling individuals who may have had undiagnosed leptospirosis.

All cases and controls under the age of 16 were excluded.

### Participant recruitment and data collection

Cases were identified as described earlier [22] to ensure all eligible cases were approached. Eligible cases were contacted and invited either by Public Health Units (if they tested positive in diagnostic laboratories) or the research team (if they tested positive in the research laboratory). All cases who agreed to participate were subsequently contacted by the research team via telephone to obtain verbal consent (Appendix 1 of the Supplementary Material) and to complete the survey involving the cases’ support network when necessary. Cases were asked about their exposures in the month before they became ill.

Contact details of the NZHS cohorts were acquired from the Ministry of Health and sent to two market research companies [22] who conducted the control surveys over the telephone. For logistical reasons, controls were contacted, consented, and interviewed in batches of 50 at six time periods during the study. Controls were asked about their exposures in the month preceding the interview [22].

### Study variables

The development of the study questionnaire is described previously [22]. The variables for this study were grouped in seven main categories, including sociodemographic factors; contact and activities with livestock; contact and activities with pets; contact and activities with wild mammals; contact or activities with any animals or their products; water and environmental exposures; and health status (Appendix 2 of the Supplementary Material).

### Data handling

Variables were either analysed individually or aggregated to reduce complexity and multicollinearity in multivariable models if deemed necessary. For example, the aggregate variable ‘any livestock contact’ was created by combining all variables related to contact with different livestock species. Activities with livestock were grouped by animal production type (dairy cattle, beef cattle, or sheep) or by activities (assisting calving and assisting lambing were included in an aggregate variable ‘assisting birth’). All activities involved in slaughtering (slaughtering, skinning, dressing carcasses, home killing, and killing for welfare reasons) were included in an aggregate variable called ‘any slaughter activities’. Any slaughter activities were also assessed by wearing gloves as PPE. Seeing evidence of rats and mice was aggregated as ‘seeing evidence of rodents’, as the distinction between rat and mouse ‘gnawings’ and droppings was considered difficult. Exposures to recreational water from creeks, lakes, streams, and dams were aggregated as ‘rivers’. Reactional activities such as swimming, boating, and fishing were aggregated as ‘any recreational water activities’.

When more than one answer was given for ethnicity, the New Zealand prioritized ethnicity was used in order of priority: Māori, Pacific Peoples, European, Asian, MELAA (Middle Eastern, Latin American, and African), and other ethnicities [25].

Occupations were grouped into six broad sectors: dairy, dry stock, meat works, mixed stock, not working (includes unemployed and retired), and other occupations (occupations that did not fall under the previous five sectors).

### Statistical analysis

All data were analysed using R version 4.3.1 [26] with packages listed in Supplementary Table S1. Statistical significance was set at *p* ≤ 0.05 for all analyses.

Descriptive statistics were used to compare the sociodemographic characteristics of cases and controls. Fisher’s exact test was used to test for associations between exposures and case status.

Individual associations were initially analysed with unconditional logistic regression that was adjusted for the matched variables to calculate partially adjusted odds ratios (paORs) and 95% confidence intervals (CIs) to inform final variable inclusion in the multivariable logistic regression (MLR) models, which were used to calculate the adjusted odds ratios (aORs).

MLR models were constructed using a bidirectional stepwise variable selection approach. Three separate regression models were used to show the impact of adjustments on the association between exposures and leptospirosis risk. Model A was a priori adjusted for only the matching variables to establish a baseline; model B included additional adjustments for age and season; and model C further included the occupational sector due to the significant association of leptospirosis with livestock occupations. Age was adjusted for because controls were significantly older than cases (Wilcoxon *p* < 0.001), and age is frequently associated with exposures and health outcomes [27]. Season was adjusted for because 50% of control interviews were conducted during summer (21 December to 20 March), compared to only 21% of cases (Table 1). Individual associations were included in the preliminary MLR model if they had paORs of ≥3 or ≤0.3, or a *p* ≤0.2 (threshold). Variables with a Pearson correlation coefficient of ≥0.5 were subsequently removed from the model by running the model with each correlated variable separately (e.g., contact with dairy cattle and activities with dairy cattle), and the variable with the lowest *p*-value was kept. The inclusion of two-level interaction terms was considered but not included due to the complexity of the model and the abundance of potential interactions. Final variable selection was performed with stepwise regression until the lowest Akaike Information Criterion was achieved. Least absolute shrinkage and selection operator (LASSO) regression [28] was used on the final variables to prioritize the importance of the risk factors while excluding those that were not strongly supported. Model fit was evaluated with five metrics: sensitivity, specificity, Area Under the Receiver Operating Characteristic curve, Cox and Snell’s Pseudo-R2, and Hosmer and Lemeshow test (Supplementary Table S2).

**Table 1.** Sociodemographic characteristics, season of exposure, and District Health Boards of 95 cases and 300 controls in a case–control study to identify risk factors for leptospirosis in Aotearoa New Zealand

Variable	Level	Case *n* (%)	Control *n* (%)	Crude odds ratios (95% CI)	*p*-value
Sex	Male	70 (73.7)	266 (88.7)	2.8 (1.5–5.2)	<0.001
	Female	25 (26.3)	34 (11.3)	Ref	
Rurality	Rural	72 (75.8)	173 (57.7)	2.3 (1.3–4.1)	0.002
	Urban	23 (24.2)	127 (42.3)	Ref	
Age (years)	16–29	15 (15.8)	21 (7.0)	6.8 (2.3–22.5)	<0.001
	30–39	18 (18.9)	25 (8.3)	6.9 (2.4–21.9)	<0.001
	40–49	15 (15.8)	61 (20.3)	2.4 (0.8–7.4)	0.1
	50–59	23 (24.2)	47 (15.7)	4.7 (1.8–14.1)	<0.001
	60–69	17 (17.9)	78 (26.0)	2.1 (0.8–6.4)	0.1
	70+	7 (7.4)	68 (22.7)	Ref	
Ethnicity^a^	Māori	13 (13.7)	39 (13.0)	1.0 (0.3–2.7)	1.0
	European	71 (74.7)	229 (76.3)	0.9 (0.4–2.1)	0.8
	Other ethnicities	11 (11.6)	32 (10.7)	Ref	
Education	No education	0	8 (2.7)	0 (0–2.5)	0.4
	Primary	5 (5.3)	3 (1.0)	6.6 (1.2–44.3)	0.01
	Secondary	52 (54.7)	136 (45.5)	1.5 (0.9–2.5)	0.09
	Tertiary	38 (40)	152 (50.8)	Ref	
Income	NZD 1 to NZD 14 000	4 (4.2)	24 (8.0)	0.8 (0.09–9.7)	1.0
	NZD 14 000 to NZD 48 000	24 (25.3)	100 (33.3)	1.1 (0.2–10.9)	1.0
	NZD 48 000 to NZD 70 000	30 (31.6)	63 (21.0)	2.1 (0.4–21.5)	0.5
	>NZD 70 000	30 (31.6)	91 (30.3)	1.5 (0.3–14.8)	1.0
	No answer	1 (1.1)	13 (4.3)	0.4 (0.005–7.9)	0.6
	No income	2 (2.1)	9 (3.0)	Ref	
Occupational sector	Dairy	22 (23.2)	11 (3.7)	15.3 (6.1–40.6)	<0.001
	Dry stock	19 (19.0)	11 (3.7)	13.2 (5.1–35.6)	<0.001
	Meat works	10 (10.5)	1 (0.3)	74.9 (9.8–3 332.4)	<0.001
	Mixed stock	13 (13.7)	20 (6.7)	5.0 (2.0–12.6)	<0.001
	Not working	11 (11.6)	101 (33.7)	0.8 (0.4–2.0)	0.8
	Other occupations	20 (21.1)	156 (52.0)	Ref	
Season^b^	Spring	34 (35.8)	51 (17.0)	2.5 (1.1–5.9)	0.01
	Summer	20 (21.1)	149 (49.7)	0.5 (0.2–1.2)	0.09
	Winter	28 (29.5)	50 (16.7)	2.1 (0.9–5.1)	0.06
	Autumn	13 (13.7)	50 (16.7)	Ref	
District Health Boards	Waikato	30 (31.6)	24 (8.0)	6.7 (1.3–68.0)	0.01
	Northland	13 (13.7)	28 (9.3)	2.5 (0.4–26.6)	0.3
	Southern	9 (9.8)	45 (15.0)	1.1 (0.2–11.9)	1.0
	Hawke’s Bay	8 (8.4)	21 (7.0)	2.1 (0.3–23.2)	0.5
	Midcentral	8 (8.4)	4 (1.3)	9.8 (1.2–134.3)	0.02
	Taranaki	7 (7.4)	7 (2.3)	5.1 (0.7–65.0)	0.1
	Bay of Plenty	6 (6.3)	17 (5.7)	1.9 (0.3–22.7)	0.7
	Whanganui	4 (4.2)	10 (3.3)	2.1 (0.2–28.5)	0.6
	Auckland	3 (3.2)	7 (2.3)	2.3 (0.2–33.8)	0.6
	Canterbury	2 (2.1)	27 (9.0)	0.4 (0.03–6.4)	0.6
	Nelson Marlborough	2 (2.1)	15 (5.0)	0.7 (0.05–11.7)	1.0
	Hutt Valley	1 (1.1)	6 (2.0)	0.9 (0.01–21.4)	1.0
	Waitemata	0	12 (4.0)	0 (0–5.7)	0.5
	West Coast	0	12 (4.0)	0 (0–5.7)	0.5
	South Canterbury	0	7 (2.3)	0 (0–10.1)	0.5
	Capital and Coast	0	16 (5.3)	0 (0–4.2)	0.2
	Wairarapa	0	10 (3.3)	0 (0–6.9)	0.5
	Counties Manukau	0	12 (4.0)	0 (0–5.7)	0.5
	Lakes	0	9 (3.0)	0 (0–7.7)	0.5
	Tairawhiti	2 (2.1)	11 (3.7)	Ref	

a There were no Pacific Peoples or MELAA cases; one Asian case was included in Other.

b Season was calculated from the date of disease onset for cases and the date of interview for controls, which corresponds to the date used to inquire about exposures in the month preceding the occurrence.

Population-attributable fractions (PAFs) were calculated with CIs computed via bootstrapping from 1 000 simulations [29].

### Ethical approval

This study received human ethics approval from the Health and Disability Ethics Committee, reference number 19/STH/80 and locality agreements, together with local Māori consultation from the 20 District Health Boards (DHBs) in Aotearoa.

## Results

### Sociodemographic characteristics of participants

Between July 2019 and January 2022, 220 cases were notified to EpiSurv [20]; of these, 139 agreed to be contacted by the research team, 131 were able to be contacted and invited to participate, 12 declined, and 24 did not meet the case definition. Thus, 43% (95/220) of cases approached over the study period met the case definition and agreed to participate in the study. A total of 1 340 controls were called; 238 had no active telephone connection, 539 could not be contacted (no answer/engaged), 61 were not eligible, and 202 did not participate (162 refused, 35 had language issues, and 5 abandoned/stopped the interview). Thus, 53% (300/563) of controls approached over the study period met the control definition and agreed to participate in the study. In total, 395 participants (95 cases and 300 controls) were interviewed between 25 July 2019 and 13 April 2022.

The percentage of males among controls (89%) was significantly higher compared with cases (74%, *p* < 0.001). A higher percentage of cases lived in rural areas (76%) compared with controls (58%, *p* = 0.002). Cases were younger (median: 49 years; interquartile range (IQR): 35.5–59.5) than controls (median: 59 years; IQR: 46–57.8) and 35% of controls were >65 years of age compared to 13% of cases (*p* < 0.001). The percentage of cases (67%) employed in the livestock industry (dairy, dry stock, mixed stock, and meat works) was significantly higher compared with controls (14.4%, *p* < 0.001). Controls were predominantly engaged in occupations that were not in the livestock industry (52%) or were not working (retired/unemployed = 33.7%, *p* < 0.001). No significant differences in ethnicity were observed between cases and controls. The sex, age, and ethnicity of cases in this study (Table 1) are reflective of all notified cases that met the Ministry of Health case definition during the study period (Supplementary Table S3).

Table 1 also shows seasonality, which corresponds to the date used to inquire about exposures in the month preceding the occurrence and the distribution of cases from the 20 DHBs. Waikato DHB had the highest proportion of cases (32%), which is consistent with notification data [30].

### Individual associations

Tables 2–5 present individual associations between potential risk factors and leptospirosis (*p* ≤ 0.2), while associations with *p* >0.2 are in Supplementary Tables S4–S9.

**Table 2.** Individual association between livestock factors and leptospirosis in Aotearoa New Zealand, adjusted for sex and rurality in logistic regression analysis (*p*-value ≤ 0.2)

Variable	Level	Case *n* (%)	Control *n* (%)	Partially adjusted odds ratios (95% CI)	*p*-value
*Had direct contact with livestock and/or their urine*					
Cattle (dairy + beef)	Yes	65 (67.4)	75 (25)	7.0 (4.0–12.6)	<0.001
	No	30 (31.6)	225 (75)	Ref	
Dairy cattle	Yes	46 (48.4)	32 (10.7)	7.2 (4.1–12.9)	<0.001
	No	49 (51.6)	268 (89.3)	Ref	
Beef cattle	Yes	43 (45.3)	61 (20.3)	3.0 (1.8–5.1)	<0.001
	No	51 (54.7)	239 (79.7)	Ref	
Sheep	Yes	34 (35.8)	70 (23.3)	1.7 (1.0–2.9)	<0.001
	No	61 (64.2)	230 (76.7)	Ref	
Pigs	Yes	17 (17.9)	20 (6.7)	3.4 (1.6–7.0)	<0.001
	No	78 (82.1)	280 (93.3)	Ref	
Any livestock contact	Yes	75 (78.9)	129 (42.7)	5.1 (2.8–9.7)	<0.001
	No	20 (21.1)	171 (40.3)	Ref	
*Did activities with livestock*					
Cattle	Yes	45 (47.4)	42 (14)	6.0 (3.4–10.7)	<0.001
	No	50 (52.6)	258 (86)	Ref	
Dairy cattle	Yes	30 (31.6)	25 (8.3)	4.4 (2.4–8.3)	<0.001
	No	65 (68.4)	275 (91.7)	Ref	
Beef cattle	Yes	25 (26.3)	25 (8.3)	4.1 (2.1–7.9)	<0.001
	No	70 (73.7)	275 (91.7)	Ref	
Sheep	Yes	25 (26.3)	43 (14.3)	1.8 (1.0–3.4)	0.05
	No	70 (73.7)	257 (85.7)	Ref	
*Were involved in livestock-related activities*					
Assisting birth	Yes	20 (21.1)	16 (5.3)	4.1 (1.9–8.6)	<0.004
	No	75 (78.9)	284 (94.7)	Ref	
Milking	Yes	24 (25.3)	15 (5.0)	5.6 (2.8–11.8)	<0.001
	No	71 (74.7)	285 (95.0)	Ref	
Castrating/docking	Yes	14 (14.7)	15 (5.0)	2.9 (1.3–6.5)	0.009
	No	81 (85.3)	285 (95.0)	Ref	
Slaughtering	Yes	35 (36.8)	41 (13.7)	3.7 (2.1–6.5)	<0.001
	No	60 (63.2)	259 (86.3)	Ref	
Slaughter/gloves	Slaughtering with gloves	13 (13.7)	9 (3.0)	6.1 (2.4–16.1)	<0.001
	Slaughtering without gloves	21 (22.1)	28 (9.3)	3.4 (1.7–6.6)	<0.001
	Missing data	1 (1.05)	4 (1.3)	0.8 (0.04–5.8)	0.8
	No slaughter activities	60 (63.2)	259 (86.3)	Ref	
Drenching	Yes	35 (36.8)	37 (12.3)	4.1 (2.3–7.5)	<0.001
	No	60 (63.2)	263 (87.7)	Ref	
Cleaning urine or faeces from yard surfaces	Yes	43 (45.3)	28 (9.3)	7.5 (4.1–13.8)	<0.001
	No	52 (54.7)	272 (90.7)	Ref	
Other activities	Yes	37 (38.9)	17 (5.7)	9.5 (5.0–18.8)	<0.001
	No	58 (61.1)	283 (94.3)	Ref	
Any livestock activities	Yes	77 (81.1)	85 (28.3)	12.9 (6.9–25.9)	<0.001
	No	18 (18.9)	215 (71.7)	Ref

**Table 3.** Individual association between pets, mammalian wildlife, and contact/activities with any animals or their products, and leptospirosis in Aotearoa New Zealand, adjusted for sex and rurality in logistic regression analysis (*p*-value ≤ 0.2)

Variable	Level	Case *n* (%)	Control *n* (%)	Partially adjusted odds ratios (95% CI)	*p*-value
*Had direct contact with pets*					
Dogs	Yes	69 (72.6)	232 (77.3)	0.6 (0.3–1.0)	0.05
	No	26 (27.4)	68 (22.7)	Ref	
Cats	Yes	50 (52.6)	183 (61)	0.6 (0.4–1.0)	0.08
	No	45 (47.4)	117 (39)	Ref	
*Cleaned up after pets*					
Dogs	Yes	21 (22.1)	95 (31.7)	0.6 (0.3–1.0)	0.06
	No	74 (77.9)	205 (68.3)	Ref	
Any pet contact or activity	Yes	78 (82.1)	270 (90.0)	0.3 (0.2–0.7)	0.004
	No	17 (17.9)	30 (10)	Ref	
*Had direct contact with wildlife*					
Rabbits	Yes	13 (13.7)	22 (7.3)	1.9 (0.9–4.1)	0.09
	No	82 (86.3)	278 (92.7)	Ref	
Goats	Yes	5 (5.3)	6 (2.0)	2.9 (0.8–10.2)	0.09
	No	90 (94.7)	294 (98.0)	Ref	
Contact with any wildlife	Yes	40 (42.1)	97 (32.3)	1.5 (0.9–2.4)	0.2
	No	55 (57.9)	203 (67.7)	Ref	
*Saw evidence of wildlife*					
Hedgehogs	Yes	4 (4.2)	22 (7.3)	0.5 (0.1–1.4)	0.2
	No	91 (95.7)	278 (92.7)	Ref	
Rats	Yes	32 (33.7)	45 (15.0)	2.6 (1.5–4.6)	<0.001
	No	63 (66.3)	255 (85.0)	Ref	
Mice	Yes	22 (23.2)	40 (13.3)	1.7 (0.9–3.1)	0.08
	No	73 (76.8)	260 (86.7)	Ref	
Rodents (rats + mice)	Yes	36 (37.9)	62 (20.7)	2.1 (1.2–3.5)	0.007
	No	59 (62.1)	238 (79.3)	Ref	
*Contact/activities with any animals or their products*					
Hunting	Yes	19 (20.0)	37 (12.3)	1.6 (0.8–3.1)	0.2
	No	76 (80.0)	263 (87.7)	Ref	
Contact with dead animals (not slaughtered on purpose)	Yes	22 (23.2)	47 (15.7)	1.5 (0.8–2.7)	0.2
	No	73 (76.8)	253 (84.3)	Ref	
Abortion/stillbirths	Yes	15 (15.8)	9 (3.0)	5.8 (2.4–14.6)	<0.001
	No	80 (84.2)	291 (97.0)	Ref	
Drank raw milk	Yes	17 (17.9)	21 (7.0)	2.6 (1.3–5.2)	0.009
	No	78 (82.1)	279 (93.0)	Ref

**Table 4.** Individual association between water/environmental exposures and leptospirosis in Aotearoa New Zealand, adjusted for sex and rurality in logistic regression analysis (*p*-value ≤ 0.2)

Variable	Level	Case *n* (%)	Control *n* (%)	Partially adjusted odds ratios (95% CI)	*p*-value
*Source of water supply at home*					
	Creek/stream	8 (8.4)	6 (2.0)	5.3 (1.5–20.0)	0.01
	Roof/rainwater	23 (24.2)	58 (19.3)	1.7 (0.7–4.1)	0.2
	Town water supply	27 (28.4)	159 (53.0)	0.7 (0.3–1.8)	0.4
	Private bore/spring	26 (27.4)	39 (13.0)	2.4 (1.0–5.8)	0.05
	Other	11 (11.6)	38 (12.7)	Ref	
*Source of water supply at work*					
	Creek/stream	14 (14.7)	5 (1.7)	26.5 (7.8–104.3)	<0.001
	Roof/rainwater	13 (13.7)	12 (4.0)	10.7 (3.7–33.6)	<0.001
	Town water supply	29 (30.5)	130 (43.3)	2.2 (1.0–5.3)	0.06
	Private bore/spring	16 (16.8)	26 (8.7)	5.0 (1.9–13.7)	0.001
	Other^a^	14 (14.7)	50 (16.7)	2.4 (0.9–6.4)	0.06
	No work water	9 (9.5)	77 (25.7)	Ref	
*Contact with water for recreational purposes*					
Ocean	Yes	17 (17.9)	104 (34.7)	0.4 (0.2–0.7)	0.002
	No	78 (82.1)	196 (65.3)	Ref	
*Participated in recreational water activities*					
Swimming	Yes	11 (11.6)	77 (25.7)	0.4 (0.2–0.7)	0.006
	No	84 (88.4)	223 (74.3)	Ref	
Boating	Yes	5 (5.3)	43 (14.3)	0.3 (0.1–0.8)	0.02
	No	90 (94.7)	257 (85.7)	Ref	
Fishing	Yes	10 (10.5)	55 (18.3)	0.5 (0.2–1.0)	0.07
	No	85 (89.5)	245 (81.7)	Ref	
Other water activities	Yes	5 (5.3)	51 (17.0)	0.3 (0.1–0.6)	0.008
	No	90 (94.7)	249 (83)	Ref	
Any recreational water activities	Yes	18 (18.9)	125 (41.7)	0.3 (0.2–0.5)	<0.001
	No	77 (81.1)	175 (58.3)	Ref	
*Encountered the following situation*					
Mud	Yes	50 (52.6)	124 (41.3)	1.5 (0.9–2.5)	0.09
	No	45 (47.4)	176 (58.7)	Ref	
Soil	Yes	50 (52.6)	227 (75.7)	0.3 (0.2–0.6)	<0.001
	No	45 (47.4)	73 (24.3)	Ref	
Walked barefoot outside	Yes	47 (49.5)	177 (59.0)	0.6 (0.4–1.0)	0.05
	No	48 (50.5)	123 (41.0)	Ref	
Hiking/walking	Yes	18 (18.9)	144 (48.0)	0.3 (0.1–0.4)	<0.001
	No	77 (81.1)	156 (52.0)	Ref	

a Unsure was added to other.

**Table 5.** Individual association between health status and leptospirosis in Aotearoa New Zealand, adjusted for sex and rurality in logistic regression analysis (*p*-value ≤ 0.2)

Variable	Level	Case *n* (%)	Control *n* (%)	Partially adjusted odds ratios (95% CI)	*p*-value
*Had cuts and scratches*					
	Uncovered cuts	59 (62.1)	96 (32.3)	3.5 (1.9–6.7)	<0.001
	Covered cuts	17 (17.9)	97 (32.3)	1.0 (0.4–1.9)	1.0
	No cuts	19 (20)	107 (35.7)	Ref	
Smoking	Smoker	12 (12.6)	34 (11.3)	0.8 (0.4–1.7)	0.6
	Ex-smoker	21 (22.1)	109 (36.3)	0.5 (0.3–0.9)	0.03
	Not a smoker	62 (65.3)	157 (52.3)	Ref	
Hay fever	Yes	19 (20.0)	100 (33.3)	0.5 (0.3–0.8)	0.01
	No	76 (80.0)	200 (66.7)	Ref	
Asthma	Yes	8 (8.4)	43 (14.3)	0.5 (0.2–1.0)	0.08
	No	87 (91.6)	257 (85.7)	Ref	
On regular medication	Yes	35 (36.8)	144 (48.0)	0.7 (0.4–1.1)	0.1
	No	60 (63.2)	156 (52.0)	Ref	

Significant risk factors included any livestock contact, specifically contact with dairy and beef cattle, sheep, and pigs, as well as activities like assisting birth, milking, castrating, drenching, cleaning urine/faeces, and slaughtering with or without gloves (Table 2). Contact with goats, deer, alpacas, horses, or poultry, and activities like shearing or trimming soiled wool (crutching) were not significantly associated (Supplementary Table S4).

Contact with pet dogs was inversely associated with leptospirosis (Table 3), while most cases had no contact with other pets (Supplementary Table S5). Seeing evidence of rats and rodents was a significant risk factor, but direct wildlife contact was not (Table 3 and Supplementary Table S6). Handling aborted materials, stillbirths, and drinking raw milk were significant risks, whereas hunting and handling animal feed/manure were not (Table 3 and Supplementary Table S6).

Work water supply from creeks/streams, rainwater, private bore/spring, and town water was significantly associated with leptospirosis, while home water supply was only significant for creeks/streams and private bore/spring (Table 4). Ocean contact, recreational water activities, soil exposure, and walking barefoot were inversely associated (Table 4), with other environmental factors non-significant (Supplementary Table S7).

Uncovered cuts/scratches were a significant risk factor, while hay fever was inversely associated (Table 5). Other common health issues were not significantly associated with leptospirosis (Supplementary Table S8).

### Multivariable risk factor analysis

Table 6 presents the aORs of three models, showing minimal variation between the baseline model A and model B, which was adjusted for confounders. Risk factors significantly associated with leptospirosis in model B included contact with dairy cattle, activities with beef cattle, cleaning urine/faeces from yard surfaces, work water supply from either creeks/streams or roof-collected rainwater, and uncovered cuts/scratches. Seeing evidence of rodents was identified as a risk factor but was excluded with LASSO regression. Significant factors inversely associated with leptospirosis included any recreational water activities, exposure to soil, and being an ex-smoker. When additionally adjusted for the occupational sector, significant risk factors retained included activities with beef cattle, cleaning urine/faeces from yard surfaces, work water supply from creeks/streams, uncovered cuts/scratches, and seeing evidence of rodents. Other risk factors identified when adjusted for occupation but not reaching statistical significance included work water supply from roof-collected rainwater and slaughtering without gloves; the latter was excluded with LASSO regression. Significant factors inversely associated with leptospirosis remained similar with any recreational water activities and exposure to soil. Pet cats, smoking, hay fever, and activities with sheep were also inversely associated with leptospirosis, all of which were excluded by LASSO regression.

**Table 6.** Multivariable association between risk factors and leptospirosis in Aotearoa New Zealand

Variable	Level	Adjusted odds ratios (95% CI)		
		Model A^a^	Model B^b^	Model C^c^
Contact with dairy cattle		3.9 (1.7–9.0)*	2.5 (1.0–6.0)*	–
Activities with beef cattle		2.8 (1.1–7.6)*	3.0 (1.1–8.2)*	4.5 (1.2–17.9)*
Cleaning urine or faeces from yard surfaces		3.5 (1.4–8.7)*	3.9 (1.5–10.3)*	3.3 (1.1–10.2)*
Work water supply	Creek/stream	7.3 (1.5–40.3)*	7.8 (1.5–45.1)*	21.2 (1.9–265.0)*
	Rain	5.3 (1.2–23.7)*	6.6 (1.4–33.7)*	6.2 (0.8–55.6)
	Town	1.5 (0.6–4.1)	1.3 (0.5–3.9)	5.4 (0.8–38.6)
	Bore/spring	0.8 (0.2–3.0)	0.8 (0.2–3.2)	1.4 (0.2–9.7)
	Other	1.2 (0.3–3.9)	1.1 (0.3–4.1)	3.3 (0.4–23.2)
	No work water	Ref	Ref	Ref
Cuts and scratches	Uncovered cut/scratch	3.9 (1.7–9.2)*	4.6 (1.9–11.7)*	9.5 (3.3–31.3)*
	Covered cut/scratch	0.6 (0.2–1.5)	0.6 (0.2–1.6)	1.1 (0.3–3.7)
	No cuts or scratches	Ref	Ref	Ref
Slaughter/gloves	Slaughtering with gloves	4.5 (1.1–19.3)*	–	0.1 (0.01–0.7)*
	Slaughtering without gloves	2.3 (0.8–6.5)	–	3.3 (0.9–12.9)
	Missing data	4.0 (0.2–41.0)	–	6.1 (0.2–75.3)
	No slaughter activities	Ref	Ref	Ref
Seeing evidence of rodents		2.5 (1.1–5.5)*	2.2 (1.0–5.0)	2.6 (1.1–6.5)*
Any recreational water activities		0.2 (0.08–0.4)*	0.3 (0.1–0.6)*	0.4 (0.1–0.9)*
Exposure to soil		0.3 (0.2–0.7)*	0.3 (0.1–0.6)*	0.3 (0.1–0.8)*
Seeing evidence of hedgehogs		0.2 (0.04–1.2)	–	–
Pet cats		–	0.5 (0.2–1.0)	0.4 (0.2–0.9)*
Smoking	Smoker	0.5 (0.2–1.3)	0.4 (0.1–1.3)	0.1 (0.03–0.5)*
	Ex-smoker	0.4 (0.2–0.9)*	0.4 (0.2–0.9)*	0.3 (0.2–0.9)*
	Not a smoker	Ref	Ref	Ref
Activities with sheep		–	–	0.2 (0.03–0.7)*
Hay fever		–		0.3 (0.1–0.9)*
On regular medication		–	–	0.5 (0.2–1.1)

a Adjusted for sex and rurality.

b Additionally adjusted for confounders’ age and season.

c Additionally adjusted for the occupational sector.

* *p*-value ≤ 0.05; greyed-out adjusted odds ratios represent variables that were excluded by least absolute shrinkage and selection operator regression.

Table 7 presents the PAFs of the three models. The highest PAFs in models B and C were for uncovered cuts, followed by seeing evidence of rodents, cleaning urine/faeces from yard surfaces, and activities with beef cattle. The PAF for different sources of work water supply was higher in model C than in model B (Table 7).

**Table 7.** Population-attributable fractions for risk factors associated with leptospirosis in Aotearoa New Zealand

Variable	Level	Population-attributable fractions (95% CI)		
		Model A^a^	Model B^b^	Model C^c^
Contact with dairy cattle		0.2 (0.07 to 0.5)*	0.1 (−0.01 to 0.4)	–
Activities with beef cattle		0.1 (−0.01 to 0.4)	0.1 (−0.001 to 0.4)	0.2 (0.01 to 0.8)*
Cleaning urine or faeces from yard surfaces		0.2 (0.01 to 0.5)*	0.2 (0.1 to 0.6)*	0.2 (−0.01 to 0.7)
Work water supply	Creek/stream	0.1 (0.002 to 0.5)*	0.1 (0.01 to 0.5)*	0.3 (0.004 to 0.9)*
	Rain	0.1 (0.01 to 0.6)*	0.2 (−0.002 to 0.7)*	0.2 (−0.04 to 0.9)*
	Town	0.2 (−0.2 to 0.6)	0.1 (−0.3 to 0.6)	0.7 (−0.3 to 0.9)*
	Bore/spring	−0.02 (−0.1 to 0.2)	−0.02 (−0.1 to 0.2)	0.03 (−0.1 to 0.6)
	Other	0.03 (−0.1 to 0.3)	0.01 (−0.2 to 0.4)	0.3 (−0.2 to 0.9)
	No work water	Ref	Ref	Ref
Cuts and scratches	Uncovered cut/scratch	0.5 (0.2 to 0.8)*	0.5 (0.2 to 0.9)*	0.7 (0.5 to 0.9)*
	Covered cut/scratch	−0.2 (−0.4 to 0.2)	−0.2 (−0.4 to 0.2)	0.03 (−0.4 to 0.7)
	No cuts or scratches	Ref	Ref	Ref
Slaughter/gloves	Slaughtering with gloves	0.1 (−0.001 to 0.4)*	–	−0.03 (−0.01 to −0.01)
	Slaughtering without gloves	0.1 (−0.05 to 0.4)	–	0.2 (−0.1 to 0.7)
	Missing data	0.04 (0.02 to 0.4)	–	0.1 (−0.02 to 0.7)
	No slaughter activities	–	–	Ref
Seeing evidence of rodents		0.2 (0.01 to 0.6)	0.2 (−0.03 to 0.6)	0.3 (−0.1 to 0.8)
Any recreational water activities		−0.5 (−0.8 to −0.3)	−0.4 (−0.7 to −0.2)	−0.4 (−0.7 to −0.02)
Exposure to soil		−0.9 (−2.1 to −0.3)	−1.0 (−2.4 to −0.4)	−1.0 (−2.7 to −0.1)
Seeing evidence of hedgehogs		−0.06 (−0.1 to −0.01)	–	–
Pet cats		–	−0.4 (−1.2 to 0.04)	−0.6 (−1.5 to −0.1)
Smoking	Smoker	−0.1 (−0.1 to 0.02)	−0.1 (−0.1 to 0.04)	−0.1 (−0.2 to −0.04)
	Ex-smoker	−0.3 (−0.5 to −0.04)	−0.3 (−0.5 to −0.1)	−0.3 (−0.6 to −0.1)
	Not a smoker	Ref	Ref	Ref
Activities with sheep		–	–	−0.1 (−0.2 to −0.01)
Hay fever		–	–	−0.3 (−0.6 to −0.01)
On regular medication		–	–	0.3 (−0.1 to 0.8)

a Adjusted for sex and rurality.

b Additionally adjusted for confounders’ age and season.

c Additionally adjusted for the occupational sector.

* *p*-value ≤ 0.05.

## Discussion

This study identified modifiable risk factors for human leptospirosis in Aotearoa, including novel behavioural and environmental factors, and confirmed its continued association with livestock occupational factors.

Uncovered cuts/scratches, the behavioural factor with the largest PAF (Table 7), increased leptospirosis risk when adjusted for the occupational sector (Table 6). This is a global risk factor for leptospirosis [31], and common in rural, manual labour-intensive sectors such as agriculture, forestry, and fishing, which have high injury rates in Aotearoa [32]. An investigation of a subset of occupational cases from this study found that, despite awareness of this risk, wound care was inconsistently practised – often due to time constraints and impracticality in work environments – and major wounds were typically addressed, while minor cuts were often neglected [33].

The lack of glove usage during slaughter activities emerged as a risk factor when adjusted for the occupational sector, while glove usage was inversely associated with leptospirosis (Table 6). Slaughtering in an abattoir involves experienced butchers, following strict protocols for using PPE [34], which may not be as rigorously adhered to in farm settings. In this study, 52% (17/33) of mixed stock workers engaged in slaughtering activities with only 24% (8/33) wearing gloves, while 82% (9/11) of meat workers were involved in slaughtering activities, with 72% (8/11) of these wearing gloves while performing these tasks. Other PPE usage was not assessed as they correlated with variables such as contact with dairy cattle (apron) or any farm activities (boots).

Contact with dairy cattle was identified as a risk factor and was heavily confounded by the occupational sector, despite high vaccination rates (99% of dairy farms) [35]. During this study period, animal vaccines in New Zealand only targeted selected serovars (Pomona, Hardjo, and Copenhageni) and excluded others (Tarassovi and Ballum), which continued to pose risks, especially to dairy farmers as most dairy herds are milked twice daily [14]. Activities with beef cattle were also confounded by occupation, likely contributing to exposure due to lower vaccination rates (beef: 18%–25%; deer: 5%–9%; sheep: <1%) [19]. Assisting calving [19] and handling cattle have been linked to increased risk in both local and international studies [36, 37], highlighting exposure to infected urine as a common pathway.

Livestock vaccination status was collected from participants with livestock contact (Supplementary Table S9), but their knowledge was not always accurate. For instance, one case mentioned a 5-in-1 vaccine, which is not available in Aotearoa. Reported vaccination practices also differed from previous studies [19, 38]. This inconsistency is unsurprising, as meat workers typically lack herd vaccination data, and farm workers may not know specific vaccine details. As a result, vaccination status was excluded from MLR models, preventing analysis of its association with leptospirosis risk.

Rodent exposure was identified as a leptospirosis risk, a novel finding for Aotearoa. Rodents, primary hosts for serovars Ballum and Copenhageni [39–41], play a key role in transmission globally, particularly in areas with poor sanitation [42]. A 2016–2017 study found high rodent densities on Aotearoa farms, with 41% shedding Ballum [39]. While most dairy cattle are vaccinated against Hardjo and Pomona (99%), only 27% are vaccinated against Copenhageni, suggesting that rodents may infect cattle with non-vaccine serovars, potentially creating a bridging host to humans [35, 43]. However, human cases associated with serovar Ballum often occur in non-livestock occupations (49%) [14], indicating that direct rodent exposure, such as farm rodent control or other outdoor, plays a larger role. While leptospirosis is historically termed ‘dairy farm fever’ in Aotearoa, it is known as ‘rat fever’ elsewhere [44], suggesting underdiagnosis among individuals with rodent exposure in non-agricultural roles [15].

Work water supply, particularly from creeks/streams, or roof-collected rainwater, was identified as a risk factor. In Aotearoa, over 10% of rural dwellings rely on untreated rainwater, with studies showing 50% of samples exceeding contamination standards and 41% heavily contaminated, often by faecal matter from animals and insects [45, 46]. Rainwater shortages during dry months lead residents to supplement with water from bores, springs, or streams, often for non-potable uses like livestock and gardening [47]. This risk likely extends to rural work water supplies, particularly on farms where home and workplace overlap. The increased risk linked to work water when adjusted for occupation suggests that infection is more likely tied to contamination during work activities, such as cleaning faeces or urine from surfaces – a known risk factor. Similar risks from natural water sources have been identified globally, with variations by region [48, 49].

Exposure to soil and recreational water activities was inversely associated with leptospirosis, suggesting that these were unlikely sources of infection in this study. While floods are a known risk factor globally [31, 50], it was not identified as a risk here due to limited exposure during the study period (Supplementary Table S7). However, mud was associated with leptospirosis (Table 4), and there was a 140% increase in leptospirosis cases in 2023 following floods. This suggests that rainfall may be a key driver for risk, leading to pathogen mobilization either through runoffs from farms or by *Leptospira* resurfacing from different soil layers during erosion [51]. Evidence of this was seen at a lake close to Auckland where leptospirosis cases were linked to swimming in the lake following flooding [52, 53]. Subsequent lake water testing detected pathogenic *Leptospira*; however, follow-up testing a month later was negative. Environmental *Leptospira* remains underexplored in Aotearoa, with only one study conducted in a farm setting [54]. While robust surveillance exists for human leptospirosis and reservoir animals [40], environmental data are lacking. Further research is needed to assess whether the environmental persistence of virulent *Leptospira* in soil and water significantly contributes to transmission, given their ability to survive in such conditions for extended periods [55].

### Limitations

During 2020–2021, leptospirosis cases (*n* = 74) averaged half of the previous 3 years (*n* = 140) [20] due to the impact of the COVID-19 pandemic. Diagnostic resources were redirected to COVID-19 testing, and overlapping symptoms with leptospirosis resulted in COVID-19 being prioritized, likely contributing to leptospirosis underdiagnosis. Telephone consultations replaced face-to-face care, potentially increasing the underreporting of milder cases. Thus, the study may have captured mostly severe cases, introducing selection bias. Furthermore, movement restrictions during the pandemic also limited recreational activities, likely reducing environmental exposure, and increased PPE use in meat processing may have lowered transmission risk in that sector, though farm risks likely remained unchanged. Lastly, the study’s PAF is most applicable to rural males, reflecting the demographic distribution of leptospirosis cases. While residual confounding may remain, these findings are crucial for shaping public health interventions for high-risk rural populations.

### Implications

One seemingly simple yet effective measure to reduce leptospirosis risk would be covering wounds, though it can be challenging in wet and dirty environments where regular plasters may not adhere. Promoting surgical or industrial-grade waterproof plasters could improve adherence in such settings [33]. This study identified contact with dairy and beef cattle as significant risk factors, with unvaccinated livestock likely posing a greater risk, as dry stock farmers and meat workers are often infected with vaccine-covered strains, while dairy farmers are infected with non-vaccine strains [14, 17], highlighting the importance of livestock vaccination. Rodent control is also crucial, with recommended measures, including baits, traps, and good hygiene practices [56]. Lastly, education and awareness campaigns are essential [57], as one-third of leptospirosis cases occurr outside the agricultural sector who are generally not aware of the disease. Collaborating with workplace experts to integrate scientific and stakeholder knowledge to help co-create practical intervention strategies for leptospirosis in Aotearoa is recommended [33].

## Conclusions

This population-based case–control study identified risk factors for human leptospirosis in Aotearoa and highlighted the complex interplay of behavioural, occupational, and environmental factors. Risk factors were associated with reservoir host animals (rodents, beef, and dairy cattle), lack of protective measures (such as gloves and wound covering), and untreated work water supply. These findings not only emphasize the need for targeted prevention efforts in Aotearoa but also offer globally relevant insights, particularly for regions where similar agricultural practices, water management challenges, and animal reservoirs drive leptospirosis risk.

## Supporting information

Nisa et al. supplementary material 1 Nisa et al. supplementary material

Nisa et al. supplementary material 2 Nisa et al. supplementary material

Nisa et al. supplementary material 3 Nisa et al. supplementary material

## Data Availability

All relevant data are provided in the tables within the main manuscript and the Supplementary Material.

## Supplementary material

The supplementary material for this article can be found at http://doi.org/10.1017/S0950268825100071.

## References

[1] Adler B and de la Pena Moctezuma A (2010) *Leptospira* and leptospirosis. Veterinary Microbiology 140(3–4), 287–296. 19345023 10.1016/j.vetmic.2009.03.012

[2] Costa F, et al. (2015) Global morbidity and mortality of leptospirosis: A systematic review. PLoS Neglected Tropical Diseases 9(9), e0003898. 26379143 10.1371/journal.pntd.0003898 PMC4574773

[3] Torgerson PR, et al. (2015) Global burden of leptospirosis: Estimated in terms of disability adjusted life years. PLoS Neglected Tropical Diseases 9(10), e0004122. 26431366 10.1371/journal.pntd.0004122 PMC4591975

[4] Haake DA and Levett PN (2015) Leptospirosis in humans. Current Topics in Microbiology and Immunology 387, 65–97. 25388133 10.1007/978-3-662-45059-8_5 PMC4442676

[5] Dunay SN, Bass JS and Stremick J (2016) Leptospirosis: A global health burden in review. Emergency Medicine 6(5), 336.

[6] Victoriano AF, et al. (2009) Leptospirosis in the Asia Pacific region. BMC Infectious Diseases 9, 147. 19732423 10.1186/1471-2334-9-147 PMC2749047

[7] Mansell C and Benschop J (2014) Leptospirosis is an important multi-species zoonotic disease in New Zealand. The New Zealand Medical Journal 127(1388), 5–8. 24481380

[8] Sanhueza JM, et al. (2020) Estimation of the burden of leptospirosis in New Zealand. Zoonoses and Public Health 67(2), 167–176. 31799801 10.1111/zph.12668

[9] *Health Act 1956* . Available at https://www.legislation.govt.nz/act/public/1956/0065/latest/whole.html (accessed 17 June 2024).

[10] Marshall R and Manktelow B (2002) Fifty years of leptospirosis research in New Zealand: A perspective. New Zealand Veterinary Journal 50, 61–63. 16032240 10.1080/00480169.2002.36270

[11] Marshall R and Chereshsky A (1996) Vaccination of dairy cattle against leptospirosis as a means of preventing human infections. Surveillance 23(1), 27–28.

[12] Fairly R (1997) Porcine leptospirosis in New Zealand. Surveillance 24(4), 15.

[13] Ministry of Primary Industries (2017) NZCP1: Design and Operation of Farm Dairies. Ministry of Primary Industries, Wellington, New Zealand.

[14] Nisa S, et al. (2020) Diverse epidemiology of *Leptospira serovars* notified in New Zealand, 1999–2017. Pathogens 9(10), 841. 33066613 10.3390/pathogens9100841 PMC7602385

[15] Thornley CN, et al. (2002) Changing epidemiology of human leptospirosis in New Zealand. Epidemiology and Infection 128(1), 29–36. 11895088 10.1017/s0950268801006392 PMC2869792

[16] Dreyfus A, et al. (2015) Risk factors for new infection with *Leptospira* in meat workers in New Zealand. Occupational and Environmental Medicine 72(3), 219–225. 25520373 10.1136/oemed-2014-102457

[17] Benschop J, Nisa S and Spencer SEF (2021) Still ‘dairy farm fever’? A Bayesian model for leptospirosis notification data in New Zealand. Journal of the Royal Society Interface 18(175), 20200964. 33593210 10.1098/rsif.2020.0964 PMC8086863

[18] Fang F, et al. (2014) Seroprevalence and exposure to risk factors for leptospirosis among veterinary students at Massey University. New Zealand Veterinary Journal 62(3), 130–135. 24350827 10.1080/00480169.2013.862161

[19] Sanhueza JM, et al. (2017) Seroprevalence and risk factors for *Leptospira* seropositivity in beef cattle, sheep and deer farmers in New Zealand. Zoonoses and Public Health 64(5), 370–380. 27918150 10.1111/zph.12317

[20] Institute of Environmental Science and Research. Available at https://www.esr.cri.nz/expertise/public-health/infectious-disease-intelligence-surveillance/ (accessed 3 October 2022).

[21] *Stats NZ* (2020) Available at https://www.stats.govt.nz/methods/urban-accessibility-methodology-and-classification (accessed 3 October 2022).

[22] Nisa S, et al. (2023) Leptospirosis in Aotearoa New Zealand: Protocol for a nationwide case–control study. JMIR Research Protocols 12, e47900. 37289491 10.2196/47900 PMC10288348

[23] Ministry of Health (2017) Available at https://www.health.govt.nz/our-work/diseases-and-conditions/communicable-disease-control-manual/leptospirosis (accessed 3 October 2022).

[24] *New Zealand Health Survey*. Available at https://www.health.govt.nz/nz-health-statistics/national-collections-and-surveys/surveys/new-zealand-health-survey (accessed 6 January 2023).

[25] *Stats NZ* (2018) Available at https://www.stats.govt.nz/tools/2018-census-ethnic-group-summaries/ (accessed 11 January 2024).

[26] R Core Team (2023) R: A language and environment for statistical computing.

[27] Grimes DA and Schulz KF (2002) Bias and causal associations in observational research. Lancet 359(9302), 248–252. 11812579 10.1016/S0140-6736(02)07451-2

[28] Friedman JH, Hastie T and Tibshirani R (2010) Regularization paths for generalized linear models via coordinate descent. Journal of Statistical Software 33(1), 1–22. 20808728 PMC2929880

[29] Kooperberg C and Petitti DB (1991) Using logistic regression to estimate the adjusted attributable risk of low birthweight in an unmatched case-control study. Epidemiology 2(5), 363–366. 1742386 10.1097/00001648-199109000-00009

[30] *Notifiable Diseases in New Zealand Annual Report 2019* (2021) Institute of Environmental Science and Research, Porirua, New Zealand, p. 12.

[31] Mwachui MA, et al. (2015) Environmental and behavioural determinants of leptospirosis transmission: A systematic review. PLoS Neglected Tropical Diseases 9(9), e0003843. 26379035 10.1371/journal.pntd.0003843 PMC4574979

[32] Kool B, et al. (2017) The epidemiology of work-related injury admissions to hospitals in the Midland region of New Zealand. Injury 48(11), 2478–2484. 28964510 10.1016/j.injury.2017.09.018

[33] Prinsen G, et al. (2024) *‘The reason why I got it* … *’ – messages from people diagnosed with leptospirosis about infection in the workplace and its impact on livelihoods*. Kōtuitui: New Zealand Journal of Social Sciences Online 19(4), 376–392

[34] *Meat Industry Association: Health and Safety Guidelines*. Available at https://www.mia.co.nz/what-we-do/workforce/health-and-safety/health-and-safety-guidelines/ (accessed 24 June 2024).

[35] Yupiana Y, et al. (2019) Emerging *Leptospira* strain poses public health risk for dairy farmers in New Zealand. Preventive Veterinary Medicine 170, 104727. 31421493 10.1016/j.prevetmed.2019.104727

[36] Agampodi SB, et al. (2015) Characteristics of rural leptospirosis patients admitted to referral hospitals during the 2008 leptospirosis outbreak in Sri Lanka: Implications for developing public health control measures. American Journal of Tropical Medicine and Hygiene 92(1), 139–144. 25331809 10.4269/ajtmh.14-0465 PMC4347370

[37] Schonning MH, et al. (2019) A case–control study of environmental and occupational risks of leptospirosis in Sri Lanka. EcoHealth 16(3), 534–543. 31664587 10.1007/s10393-019-01448-w

[38] Fang F, et al. (2015) Shedding and seroprevalence of pathogenic *Leptospira* spp. in sheep and cattle at a New Zealand abattoir. Zoonoses and Public Health 62(4), 258–268. 25043226 10.1111/zph.12146

[39] Moinet M, et al. (2023) A cross-sectional investigation of *Leptospira* at the wildlife–livestock interface in New Zealand. PLoS Neglected Tropical Diseases 17(9), e0011624. 37672535 10.1371/journal.pntd.0011624 PMC10506710

[40] Wilkinson DA, et al. (2024) Molecular typing of *Leptospira* spp. in farmed and wild mammals reveals new host–serovar associations in New Zealand. New Zealand Veterinary Journal 72(1), 1–21. 37589061 10.1080/00480169.2023.2248930

[41] Hathaway SC and Blackmore DK (1981) Ecological aspects of the epidemiology of infection with leptospires of the Ballum serogroup in the black rat (*Rattus rattus*) and the brown rat (*Rattus norvegicus*) in New Zealand. The Journal of Hygiene 87(3), 427–436. 7310125 10.1017/s0022172400069679 PMC2134120

[42] Goarant C (2016) Leptospirosis: Risk factors and management challenges in developing countries. Research and Reports in Tropical Medicine 7, 49–62. 30050339 10.2147/RRTM.S102543 PMC6028063

[43] Moinet M, et al. (2021) Of mice, cattle, and men: A review of the eco-epidemiology of *Leptospira borgpetersenii* serovar Ballum. Tropical Medicine and Infectious Disease 6(4), 189. 34698305 10.3390/tropicalmed6040189 PMC8544700

[44] Masali KA, et al. (2007) Control and prevention of rat fever (leptospirosis) outbreak in six villages of Raichur district, Karnataka. Journal of Indian Medical Association 105(11), 632, 634-6. 18405089

[45] Abbott S, Caughley BP and Douwes J (2007) The Microbiological Quality of Roof-collected Rainwater of Private Dwellings in New Zealand. In Water 2006 International Conference from Institute of Food Nutrition and Human Health, Massey University, New Zealand.

[46] Simmons G, et al. (2001) Contamination of potable roof-collected rainwater in Auckland, New Zealand. Water Research 35(6), 1518–1524. 11317899 10.1016/s0043-1354(00)00420-6

[47] Household Water Supplies (2021) Institute of Environmental Science and Research, Ministry of Health, Porirua, New Zealand.

[48] Meny P, et al. (2019) Seroprevalence of leptospirosis in human groups at risk due to environmental, labor or social conditions. Revista Argentina de Microbiología 51(4), 324–333. 30979517 10.1016/j.ram.2019.01.005

[49] Sohail ML, et al. (2018) Seroprevalence and risk factor analysis of human leptospirosis in distinct climatic regions of Pakistan. Acta Tropica 181, 79–83. 29407239 10.1016/j.actatropica.2018.01.021

[50] Baharom M, et al. (2024) Environmental and occupational factors associated with leptospirosis: A systematic review. Heliyon 10(1), e23473. 38173528 10.1016/j.heliyon.2023.e23473 PMC10761560

[51] Thibeaux R, et al. (2024) Rainfall-driven resuspension of pathogenic *Leptospira* in a leptospirosis hotspot. Science of the Total Environment 911, 168700. 37992819 10.1016/j.scitotenv.2023.168700

[52] *Our Auckland: Public Health Warning Lifted for Lake Wainamu* (2023) accessed 25 May 2024 Available at https://ourauckland.aucklandcouncil.govt.nz/news/2023/10/public-health-warning-lifted-for-lake-wainamu/.

[53] National Institute of Water and Atmospheric Research Limited. accessed 25 May 2024 Available at https://niwa.co.nz/climate-summaries/annual/annual-climate-summary-2023.

[54] Wilkinson DA, et al. (2021) Identification of pathogenic *Leptospira* species and serovars in New Zealand using metabarcoding. PLoS One 16(9), e0257971. 34587213 10.1371/journal.pone.0257971 PMC8480790

[55] Bierque E, et al. (2020) *Leptospira interrogans* retains direct virulence after long starvation in water. Current Microbiology 77(10), 3035–3043. 32683468 10.1007/s00284-020-02128-7

[56] *Trapping Project Puts Dent in Pest Population* (n.d.) Zoetis New Zealand Limited. Available at https://www.zoetis.co.nz/livestocksolutions/dairywellness/seasonal-spotlight/articles/trapping-project-puts-dent-in-pest-population.aspx (accessed 24 May 2024).

[57] Pasquier U, et al. (2020) We can’t do it on our own!’ – integrating stakeholder and scientific knowledge of future flood risk to inform climate change adaptation planning in a coastal region. Environmental Science & Policy 103, 50–57.

