# Supplementary material for: Case–control study of leptospirosis in Aotearoa New Zealand reveals behavioural, occupational, and environmental risk factors
Source: Epidemiol Infect. 2025 Jun 2;153:e67. doi: 10.1017/S0950268825100071 (PMC12171903; doi:10.1017/S0950268825100071)
Supplement: Nisa et al. supplementary material 3 — Nisa et al. supplementary material [file S0950268825100071sup003.docx]

# **Case-control study of leptospirosis in Aotearoa New Zealand reveals behavioural, occupational, and environmental risk factors** Shahista Nisa^1*^, Enrico Ortolani^1^, Emilie Vallee^2^, Jonathan Marshall^3^, Julie Collins-Emerson^1^, Polly Yeung^4^, Gerard Prinsen^5^, Jackie Wright^6^, Tanya Quin^7^, Ahmed Fayaz^1^, Stuart Littlejohn^1^, Michael G Baker^8^, Jeroen Douwes^9^, Jackie Benschop^1^

^1^Molecular Epidemiology and Public Health Laboratory, School of Veterinary Science, Massey University, Palmerston North, New Zealand

^2^EpiCentre, School of Veterinary Science, Massey University, Palmerston North, New Zealand

^3^School of Mathematical and Computational Sciences, Massey University, Palmerston North,

New Zealand

^4^School of Social Work, Massey University, Palmerston North, New Zealand

^5^School of People, Environment and Planning, Massey University, Palmerston North, New Zealand

^6^Institute of Environmental Science and Research, Christchurch, New Zealand

^7^Rural Health Unit, University of Auckland, Auckland, New Zealand

^8^Department of Public Health, University of Otago, Wellington, New Zealand

^9^Centre for Public Health Research, Massey University, Wellington, New Zealand

*Corresponding author

Email: [s.nisa@massey.ac.nz](mailto:s.nisa@massey.ac.nz) (SN)

Short title: Case-control study of leptospirosis in Aotearoa New Zealand

### Table S1: R packages

| Package name | Reference |
| --- | --- |
| RODBC | [1] |
| sqldf | [2] |
| tidyverse | [3] |
| epiR | [4] |
| descr | [5] |
| gmodels | [6] |
| lmtest | [7] |
| pROC | [8] |
| pscl | [9, 10] |
| glmnet | [11-13] |
| grateful | [14] |

### Table S2: Model evaluation output

|  | Model A | Model B | Model C |
| --- | --- | --- | --- |
| AIC | 297.24 | 286.89 | 263.08 |
| Sensitivity | 0.6 | 0.6 | 0.73 |
| Specificity | 0.95 | 0.95 | 0.97 |
| Area Under the Receiver Operating Characteristic curve | 0.9 | 0.91 | 0.94 |
| Snell's Pseudo-R2 | 0.55 | 0.58 | 0.68 |
| Hosmer and Lemeshow goodness of fit | 0.9 | 0.74 | 0.42 |

### Table S3: Demographic characteristics of notified leptospirosis cases in Aotearoa New Zealand from 2018 to 2023*

|  |  | 2018  n (%) | 2019  n (%) | 2020  n (%) | 2021  n (%) | 2022  n (%) | 2023  n (%) |
| --- | --- | --- | --- | --- | --- | --- | --- |
| Sex | Male | 95 (87.5) | 75 (84.3) | 44 (73.3) | 69 (78.4) | 100 (82.6) | 142 (83.5) |
|  | Female | 14 (12.8) | 14 (15.7) | 16 (26.7) | 19 (21.6) | 21 (17.4) | 28 (16.5) |
| Age (years) | 16-29 | 27 (24.8) | 26 (29.2) | 9 (15.0) | 15 (17.0) | 25 (20.7) | 26 (15.3) |
|  | 30-39 | 18 (16.5) | 15 (16.9) | 9 (15.0) | 23 (26.1) | 16 (13.2) | 27 (15.9) |
|  | 40-49 | 22 (20.2) | 20 (22.5) | 9 (15.0) | 11 (12.5) | 20 (16.5) | 35 (20.6) |
|  | 50-59 | 21 (19.3) | 14 (15.7) | 20 (33.3) | 16 (18.2) | 24 (19.8) | 36 (21.2) |
|  | 60-69 | 18 (16.5) | 13 (14.6) | 12 (20.0) | 8 (9.1) | 22 (18.2) | 27 (15.9) |
|  | 70+ | 3 (2.8) | 6 (6.7) | 1 (1.7) | 14 (15.9) | 13 (10.7) | 17 (10.0) |
| Ethnicity^a^ | Māori | 18 (16.5) | 11 (12.5) | 8 (13.3) | 20 (22.7) | 24 (19.8) | 28 (16.5) |
|  | European | 84 (77.1) | 72 (81.8) | 50 (83.3) | 66 (75.0) | 90 (74.4) | 126 (74.1) |
|  | Other | 7 (6.4) | 5 (5.7) | 2 (3.3) | 2 (2.3) | 7 (5.8) | 16 (9.4) |
| Total number of cases (N) |  | 109 | 89 | 60 | 88 | 121 | 170 |

*Publicly available data from EpiSurv [15]

### Table S4: Individual association between livestock factors and leptospirosis in Aotearoa New Zealand, adjusted for sex and rurality in logistic regression analysis.

| Exposure | Level | Case  n (%) | Control  n (%) | Partially adjusted ORs  (95% CI) | p-value |
| --- | --- | --- | --- | --- | --- |
| **Had direct contact with the following livestock and/or their urine** | | | | | |
| Goats | Yes | 7 (7.4) | 19 (6.3) | 1.1 (0.4-2.7) | 0.8 |
|  | No | 88 (92.6) | 281 (93.7) | Ref |  |
| Deer | Yes | 3 (3.2) | 9 (3.0) | 1.2 (0.2-4.1) | 0.8 |
|  | No | 92 (96.85) | 291 (97) | Ref |  |
| Alpacas | Yes | 0 | 2 (0.7) | <0.001 (0- >1000) | 1.0 |
|  | No | 95 (100) | 298 (99.3) | Ref |  |
| Horses | Yes | 15 (15.8) | 42 (14.0) | 0.8 (0.4-1.6) | 0.6 |
|  | No | 80 (84.2) | 258 (86.0) | Ref |  |
| Poultry | Yes | 5 (5.3) | 14 (4.7) | 0.9 (0.3-2.4) | 0.8 |
|  | No | 90 (94.7) | 286 (95.3) | Ref |  |
| **Were involved in the following livestock associated activities** | | | | | |
| Crutching | Yes | 14 (14.7) | 32 (10.7) | 1.2 (0.6-2.5) | 0.6 |
|  | No | 81 (85.3) | 268 (89.3) | Ref |  |
| Shearing | Yes | 3 (3.2) | 16 (5.3) | 0.5 (0.1-1.6) | 0.3 |
|  | No | 92 (96.8) | 284 (94.7) | Ref |  |
| **Used PPE when handling livestock^a^** | | | | | |
| Gloves | Yes | 32 (38.1) | 19 (17.3) | 2.6 (1.3-5.1) | 0.007 |
|  | No | 52 (61.9) | 91 (82.7) | Ref |  |
| Boots | Yes | 68 (81.0) | 45 (49.5) | 6.8 (3.5-14.2) | <0.001 |
|  | No | 16 (19) | 65 (59.1) | Ref |  |
| Apron | Yes | 31 (36.9) | 11 (10) | 4.9 (2.3-11.1) | <0.001 |
|  | No | 53 (63.1) | 99 (90) | Ref |  |
| Goggles | Yes | 12 (14.3) | 10 (9.1) | 1.4 (0.5-3.6) | 0.5 |
|  | No | 72 (85.7) | 100 (90.9) | Ref |  |
| Mask | Yes | 5 (6.0) | 5 (4.5) | 1.2 (0.3-4.6) | 0.8 |
|  | No | 79 (94) | 105 (95.5) | Ref |  |
| Other PPE | Yes | 15 (17.9) | 9 (8.2) | 2.3 (0.9-5.8) | 0.7 |
|  | No | 69 (82.1) | 101 (91.8) | Ref |  |
| Any PPE | Yes | 82 (97.6) | 63 (57.3) | 31.3 (8.9-199.7) | <0.001 |
|  | No | 2 (2.4) | 47 (42.8) | Ref |  |

^a^All risk factors were calculated with 95 cases and 300 controls, except for the use of PPE when handling livestock. These were only calculated with people who had exposure to livestock which included 84 cases and 110 controls.

### Table S5: Individual association between pets and leptospirosis in Aotearoa New Zealand, adjusted for sex and rurality in logistic regression analysis.

| Exposure | Level | Case  n (%) | Control  n (%) | Partially adjusted ORs  (95% CI) | p-value |
| --- | --- | --- | --- | --- | --- |
| **Had direct contact with pets** | | | | | |
| Rats | Yes | 0 | 1 (0.3) | <0.001 (0 ->1000) | 1.0 |
|  | No | 95 (100) | 299 (99.7) | Ref |  |
| Mice | Yes | 0 | 1 (0.3) | <0.001 (0 ->1000) | 1.0 |
|  | No | 95 (100) | 299 (99.7) | Ref |  |
| Guinea pigs | Yes | 0 | 6 (2.0) | <0.001 (0 ->1000) | 1.0 |
|  | No | 95 (100) | 294 (98.0) | Ref |  |
| Rabbits | Yes | 2 (2.1) | 8 (2.7) | 0.7 (0.1-2.9) | 0.6 |
|  | No | 93 (97.9) | 292 (97.3) | Ref |  |
| Other pets | Yes | 3 (3.2) | 12 (4.0) | 0.6 (0.1-2.0) | 0.4 |
|  | No | 92 (96.8) | 288 (96.0) | Ref |  |
| **Cleaned up after pets** | | | | | |
| Cats | No | 7 (7.4) | 34 (11.3) | 0.5 (0.2-1.2) | 0.1 |
|  | Yes | 88 (92.6) | 266 (88.7) | Ref |  |
| Rats | No | 0 | 1 (0.3) | <0.001 (0 ->1000) | 1.0 |
|  | Yes | 95 (100) | 299 (99.7) | Ref |  |
| Mice | No | 0 | 3 (1.0) | <0.001 (0 ->1000) | 1.0 |
|  | Yes | 95 (100) | 297 (99.0) | Ref |  |
| Guinea pigs | No | 0 | 4 (1.3) | <0.001 (0 ->1000) | 1.0 |
|  | Yes | 95 (100) | 296 (98.7) | Ref |  |
| Rabbits | No | 0 | 2 (0.7) | <0.001 (0 ->1000) | 1.0 |
|  | Yes | 95 (100) | 298 (99.3) | Ref |  |
| Other pets | No | 2 (2.1) | 10 (3.3) | 0.4 (0.07-1.8) | 0.3 |
|  | Yes | 93 (97.9) | 290 (96.7) | Ref |  |

### Table S6: Individual association between mammalian wildlife, and contact/activities with any animals or their products, and leptospirosis in Aotearoa New Zealand, adjusted for sex and rurality in logistic regression analysis.

| Variable | Level | Case  n (%) | Control  n (%) | Partially adjusted ORs  (95% CI) | p-value |
| --- | --- | --- | --- | --- | --- |
| **Had direct contact with the following wildlife** | | | | | |
| Possums | Yes | 9 (9.5) | 33 (11.0) | 0.7 (0.3-1.6) | 0.5 |
|  | No | 86 (90.5) | 267 (89.0) | Ref |  |
| Pigs | Yes | 3 (3.2) | 11 (3.7) | 0.9 (0.2-3.0) | 0.8 |
|  | No | 92 (96.8) | 289 (96.3) | Ref |  |
| Hedgehogs | Yes | 5 (5.3) | 20 (6.7) | 0.8 (0.2-2.0) | 0.6 |
|  | No | 90 (94.7) | 280 (93.3) | Ref |  |
| Rats | Yes | 12 (12.6) | 30 (10.0) | 1.3 (0.6-2.7) | 0.5 |
|  | No | 83 (87.4) | 270 (90.0) | Ref |  |
| Mice | Yes | 14 (14.7) | 32 (10.7) | 1.2 (0.6-2.4) | 0.6 |
|  | No | 81 (85.3) | 268 (89.3) | Ref |  |
| Ferrets | Yes | 0 | 3 (1.0) | <0.001 (0 ->1000) | 1.0 |
|  | No | 95 (100) | 297 (99.0) | Ref |  |
| Deer | Yes | 5 (5.3) | 14 (4.7) | 1.3 (0.4-3.5) | 0.7 |
|  | No | 90 (94.7) | 286 (95.3) | Ref |  |
| Cats | Yes | 3 (3.2) | 10 (3.3) | 0.9 (0.2-3.1) | 0.9 |
|  | No | 92 (96.8) | 290 (96.7) | Ref |  |
| Rodent (rats + mice) | Yes | 16 (16.8) | 49 (16.3) | 0.9 (0.5-1.7) | 0.8 |
|  | No | 79 (81.3) | 251 (83.7) | Ref |  |
| Other wildlife | Yes | 4 (4.2) | 11 (3.7) | 1.1 (0.3-3.4) | 0.9 |
|  | No | 91 (95.8) | 289 (96.3) | Ref |  |
| **Saw evidence of the following wildlife** | | | | | |
| Possums | Yes | 18 (18.9) | 47 (15.7) | 1.0 (0.5-1.9) | 0.9 |
|  | No | 77 (81.1) | 253 (84.3) | Ref |  |
| Pigs | Yes | 4 (4.2) | 7 (2.3) | 2.0 (0.5-6.8) | 0.3 |
|  | No | 91 (95.8) | 293 (97.7) | Ref |  |
| Ferrets | Yes | 2 (2.1) | 3 (1.0) | 2.3 (0.3-14.5) | 0.4 |
|  | No | 93 (97.9) | 297 (99.0) | Ref |  |
| Rabbits | Yes | 14 (14.7) | 52 (17.3) | 0.7 (0.4-1.4) | 0.4 |
|  | No | 81 (85.3) | 248 (82.07) | Ref |  |
| Deer | Yes | 6 (6.3) | 21 (7.0) | 0.9 (0.3-2.2) | 0.8 |
|  | No | 89 (93.7) | 279 (93.0) | Ref |  |
| Goats | Yes | 3 (3.2) | 7 (2.3) | 1.3 (0.3-5.0) | 0.7 |
|  | No | 92 (96.8) | 293 (97.7) | Ref |  |
| Cats | Yes | 6 (6.3) | 13 (4.3) | 1.2 (0.4-3.3) | 0.7 |
|  | No | 89 (93.7) | 287 (95.7) | Ref |  |
| Other wildlife | Yes | 5 (5.3) | 14 (4.7) | 1.3 (0.4-3.6) | 0.7 |
|  | No | 90 (94.7) | 286 (95.3) | Ref |  |
| Any wildlife | Yes | 48 (50.3) | 123 (41.0) | 1.2 (0.8-2.0) | 0.4 |
|  | No | 47 (47.5) | 177 (59.0) | Ref |  |
| **Handled traps for wildlife** | | | | | |
| Set traps for any wildlife | Yes | 34 (35.8) | 97 (32.3) | 1.0 (0.6-1.6) | 0.9 |
|  | No | 61 (64.2) | 203 (67.7) | Ref |  |
| Emptied traps for any wildlife | Yes | 20 (21.1) | 52 (17.3) | 1.1 (0.6-2.0) | 0.7 |
|  | No | 75 (78.9) | 248 (82.7) | Ref |  |
| Any trap activity | Yes | 35 (36.8) | 98 (32.7) | 1.0 (0.6-1.7) | 1.0 |
|  | No | 60 (63.2) | 202 (67.3) | Ref |  |
| **Used PPE while handling wildlife^a^** | | | | | |
| Gloves | Yes | 15 (28.8) | 32 (24.2) | 1.0 (0.5-2.2) | 0.9 |
|  | No | 37 (71.1) | 100 (75.5) | Ref |  |
| Boots | Yes | 24 (46.2) | 38 (28.8) | 2.5 (1.2-5.2) | 0.01 |
|  | No | 28 (53.8) | 94 (71.2) | Ref |  |
| Apron | Yes | 1 (1.9) | 1 (0.8) | 4.1 (0.2-109.4) | 0.3 |
|  | No | 51 (98.1) | 131 (99.2) | Ref |  |
| Goggles | Yes | 1 (1.9) | 2 (1.5) | 1.8 (0.1-19.8) | 0.6 |
|  | No | 51 (98.1) | 130 (98.5) | Ref |  |
| Mask | Yes | 0 | 2 (1.5) | <0.001 (0 ->1000) | 1.0 |
|  | No | 50 (100) | 130 (98.5 | Ref |  |
| Others | Yes | 4 (7.7) | 5 (3.8) | 2.2 (0.5-9.0) | 0.3 |
|  | No | 48 (92.3) | 127 (96.2) | Ref |  |
| Any PPE | Yes | 33 (63.5) | 62 (47.0) | 1.9 (0.9-3.8) | 0.08 |
|  | No | 19 (36.5) | 70 (53.0) | Ref |  |
| **Contact/activities with any animals or their products** | | | | | |
| Handled animal feed | Yes | 56 (58.9) | 151 (50.3) | 1.1 (0.7-1.8) | 0.8 |
|  | No | 39 (41.1) | 149 (49.7) | Ref |  |
| Used animal manure | Yes | 13 (13.7) | 48 (16.0) | 0.8 (0.4-1.5) | 0.5 |
|  | No | 82 (86.3) | 252 (84.0) | Ref |  |

^a^All risk factors were calculated with 95 cases and 300 controls, except for the use of PPE when handling wildlife. This was only calculated with people who had exposure to wildlife which included 52 cases and 132 controls.

### Table S7: Individual association between water/environmental exposures and leptospirosis in Aotearoa New Zealand, adjusted for sex and rurality in logistic regression analysis.

| Variable | Level | Case n (%) | Control n (%) | Partially adjusted ORs  (95% CI) | p-value |
| --- | --- | --- | --- | --- | --- |
| **Contact with water for recreational purpose** | | | | | |
| River | Yes | 28 (29.5) | 95 (31.7) | 0.8 (0.5-1.4) | 0.5 |
|  | No | 67 (70.5) | 205 (68.3) | Ref |  |
| **Encountered the following wet situation** | | | | | |
| Flooding | Yes | 7 (7.4) | 13 (4.3) | 1.7 (0.6-4.4) | 0.3 |
|  | No | 88 (92.6) | 287 (95.7) | Ref |  |
| Animal effluent | Yes | 23 (24.2) | 55 (18.3) | 1.3 (0.7-2.3) | 0.4 |
|  | No | 72 (75.8) | 245 (81.7) | Ref |  |
| Drainage/plumbing work | Yes | 11 (11.6) | 41 (13.7) | 0.9 (0.4-1.9) | 0.9 |
|  | No | 84 (88.4) | 259 (86.3) | Ref |  |
| Landscaping | Yes | 16 (16.8) | 58 (19.3) | 0.9 (0.4-1.6) | 0.6 |
|  | No | 79 (83.2) | 242 (80.7) | Ref |  |
| Wetlands | Yes | 9 (9.5) | 41 (13.7) | 0.6 (0.3-1.3) | 0.3 |
|  | No | 86 (90.5) | 259 (86.3) | Ref |  |
| Any wet situation | Yes | 62 (65.3) | 170 (56.7) | 1.3 (0.8-2.2) | 0.3 |
|  | No | 33 (34.7) | 130 (43.3) | Ref |  |
| Travelled overseas^a^ | Yes | 3 (3.2) | 4 (1.3) | 2.7 (0.5-13.6) | 0.2 |
|  | No | 92 (96.8) | 296 (98.7) | Ref |  |
| Camping | Yes | 5 (5.3) | 18 (6.0) | 0.7 (0.2-1.9) | 0.5 |
|  | No | 90 (94.7) | 282 (94) |  |  |
| **Used specific PPE while handling soil^b^** | | | | | |
| Gloves | Yes | 17 (34.0) | 112 (49.3) | 0.5 (0.2-1.0) | 0.04 |
|  | No | 33 (66.0) | 115 (50.7) | Ref |  |
| Boots | Yes | 39 (78.0) | 138 (60.8) | 2.1 (1.0-4.7) | 0.05 |
|  | No | 11 (22.0) | 89 (39.1) | Ref |  |
| Apron | Yes | 1 (2.0) | 0 | >1000 (<0.001- 0) | 1.0 |
|  | No | 49 (98.0) | 227 (100.0) | Ref |  |
| Goggles | Yes | 3 (6.0) | 10 (4.4) | 1.6 (0.3-6.0) | 0.5 |
|  | No | 47 (94.0) | 217 (95.6) | Ref |  |
| Mask | Yes | 2 (4.0) | 19 (8.4) | 0.7 (0.1-2.7) | 0.6 |
|  | No | 48 (96.0) | 208 (91.6) | Ref |  |
| Used other PPE | Yes | 4 (8.0) | 11 (4.8) | 2.4 (0.6-7.7) | 0.2 |
|  | No | 46 (92.0) | 216 (95.2) | Ref |  |
| Used any PPE | Yes | 42 (84.0) | 179 (78.9) | 1.3 (0.6-3.3) | 0.5 |
|  | No | 8 (16.0) | 48 (21.1) | Ref |  |

^a^International borders were closed from 19 March 2020 to 31 July 2022 due to the COVID-19 pandemic thus this was not considered in the MLR models.

^b^All risk factors were calculated with 95 cases and 300 controls, except for the use of PPE when handling soil. This was only calculated with people who had exposure to soil which included 50 cases and 227 controls.

### Table S8: Individual association between health status and leptospirosis in Aotearoa New Zealand, adjusted for sex and rurality in logistic regression analysis.

| Variable | Level | Case  n (%) | Control  n (%) | Partially adjusted ORs  (95% CI) | p-value |
| --- | --- | --- | --- | --- | --- |
| Diabetes | Yes | 5 (5.3) | 20 (6.7) | 0.9 (0.3-2.3) | 0.8 |
|  | No | 90 (91.8) | 278 (92.7) | Ref |  |
| Heart disease | Yes | 5 (5.3) | 29 (9.7) | 0.7 (0.2-1.7) | 0.4 |
|  | No | 90 (91.8) | 269 (89.7) | Ref |  |
| Lung disease | Yes | 0 | 7 (2.3) | <0.001 (0->1000) | 1.0 |
|  | No | 95 (100.0) | 292 (97.3) | Ref |  |
| Anxiety | Yes | 12 (12.6) | 32 (10.7) | 1.0 (0.5-2.1) | 1.0 |
|  | No | 83 (87.4) | 265 (88.3) | Ref |  |
| Depression | Yes | 12 (12.6) | 28 (9.3) | 1.0 (0.4-2.1) | 1.0 |
|  | No | 83 (87.4) | 268 (89.8) | Ref |  |
| Other health condition | Yes | 12 (12.6) | 0 | >1000 (<0.001- 0) | 1.0 |
|  | No | 83 (87.4) | 300 (100.0) | Ref |  |
| Had antibiotics | Yes | 4 (4.2) | 27 (9.0) | 0.5 (0.1-1.4) | 0.2 |
|  | No | 91 (95.8) | 273 (91.0) | Ref |  |
| Has high blood pressure | Yes | 8 (8.4) | 0 | >1000 (<0.001- 0) | 1.0 |
|  | No | 87 | 300 (100.0) | Ref |  |
| Has/had cancer | Yes | 4 (4.2) | 0 | >1000 (<0.001- 0) | 1.0 |
|  | No | 91 (95.8) | 300 (100.0 | Ref |  |

### Table S9: Individual association between vaccination status^a^ of livestock species and leptospirosis in Aotearoa New Zealand, adjusted for sex only^b^ in logistic regression analysis, p-value ≤0.2.

| Variable | Level | Case  n (%) | Control  n (%) | Partially adjusted ORs  (95% CI) | p-value |
| --- | --- | --- | --- | --- | --- |
| Dairy cattle | Fully | 34 (61.8) | 11 (61.1) | 1.9 (0.4-1.4) | 0.5 |
|  | Partially | 11 (20.0) | 3 (16.6) | 1.3 (0.1-0.2) | 0.8 |
|  | Unsure | 1 (1.8) | 2 (11.1) | 8 (0.5-24) | 0.1 |
|  | Not at all | 9 (16.4) | 2 (11.1) | Ref |  |
| Beef cattle | Fully | 16 (44.4) | 14 (46.6) | 1.2 (0.4-3.7) | 0.7 |
|  | Partially | 1 (2.8) | 1 (3.3) | 1.3 (0.05-35.7) | 0.9 |
|  | Not at all | 14 (38.8) | 10 (33.3) | 1.3 (0.3-5.9) | 0.7 |
|  | Unsure | 5 (13.8) | 5 (16.7) | Ref |  |
| Sheep | Fully | 33 (49.2) | 36 (26.7) | 0.6 (0.3-1.3) | 0.2 |
|  | Partially | 1 (1.5) | 27 (20.0) | 11.9 (2.3-218.4) | 0.02 |
|  | Not at all | 28 (41.8) | 60 (44.4) | 1.2 (0.4-4.4) | 0.7 |
|  | Unsure | 3 (4.4) | 12 (8.8) | Ref |  |
| Pigs | Fully | 3 (75.0) | 2 (50.0) | 0.6 (0.3-1.3) | 0.2 |
|  | Partially | 0 | 1 (25.0) | 11.9 (2.3-218.4) | 0.02 |
|  | Not at all | 1 (25.0) | 1 (25.0) | 1.2 (0.4-4.4) | 0.7 |
|  | Unsure | 0 | 0 | Ref |  |

^a^Vaccination status of livestock species were only calculated with people who did activities with these animals and who answered the vaccination question. Case livestock contact/activities included 55 dairy cattle, 36 beef cattle, 67 sheep, and 4 pigs while controls included 18 dairy cattle, 30 beef cattle, 135 sheep, and 4 pigs.

^b^All participants were based rurality and therefore was not adjusted.

# **References**

1. Ripley, B., and Michael Lapsley., *RODBC: ODBC Database Access*. 2023.

2. Grothendieck, G., *sqldf: Manipulate r Data Frames Using SQL.* 2017.

3. Wickham, H., et al., *Welcome to the Tidyverse.* Journal of Open Source Software, 2019. **4**: p. 1686.

4. Mark Stevenson, E.S., Telmo Nunes, Cord Heuer, Jonathon Marshall, Javier Sanchez, Ron Thorn-ton, Jeno Reiczigel, Jim Robison-Cox, Paola Sebastiani, Peter Solymos, Kazuki Yoshida, Geoff Jones, Sarah Pirikahu, Simon Firestone, Ryan Kyle, Johann Popp, Mathew Jay, Charles Reynard, Allison Cheung, Nagendra Singanallur, Aniko Szabo, Ahmad Rabiee., *epiR: Tools for the Analysis of Epidemiological Data. R package version 2.0.19.* Available from: <https://CRAN.R-project.org/package=epiR>, 2021.

5. Dirk Enzmann, J.A.I., *descr: Descriptive Statistics.* 2023.

6. Warnes, G.R., Ben Bolker, Thomas Lumley, Randall C Johnson, *gmodels: Various r Programming Tools for Model Fitting.* 2022.

7. Zeileis, A., and Torsten Hothorn., *Diagnostic Checking in Regression Relationships*. 2022.

8. Robin, X., et al., *pROC: an open-source package for R and S+ to analyze and compare ROC curves.* BMC Bioinformatics, 2011. **12**(1): p. 77.

9. Zeileis, A., C. Kleiber, and S. Jackman, *Regression Models for Count Data in R.* Journal of Statistical Software, 2008. **27**(8): p. 1 - 25.

10. Jackman, S., *pscl: Classes and Methods for R Developed in the Political Science Computational Laboratory*. 2020.

11. Friedman, J., T. Hastie, and R. Tibshirani, *Regularization Paths for Generalized Linear Models via Coordinate Descent.* Journal of Statistical Software, 2010. **33**(1): p. 1-22.

12. Simon, N., et al., *Regularization Paths for Cox's Proportional Hazards Model via Coordinate Descent.* Journal of Statistical Software, 2011. **39**(5): p. 1-13.

13. Tay, J.K., B. Narasimhan, and T. Hastie, *Elastic Net Regularization Paths for All Generalized Linear Models.* Journal of Statistical Software, 2023. **106**.

14. Francisco Rodriguez-Sanchez, C.P.J., Shaurita D. Hutchins, James M. Clawson, *grateful: Facilitate Citation of R Packages*. 2023.

15. *Institute of Environmental Science and Research*. [cited 2022 October 3]; Available from: <https://www.esr.cri.nz/expertise/public-health/infectious-disease-intelligence-surveillance/>.
